# Serum C‐Terminal Agrin Fragment With Acute and Long‐Term Exercise and Angiotensin II Type I Receptor Blockade

**DOI:** 10.1002/jcsm.13832

**Published:** 2025-06-04

**Authors:** Casper Soendenbroe, Mette F. Heisterberg, Jesper L. Andersen, Michael Kjaer, Abigail L. Mackey

**Affiliations:** ^1^ Institute of Sports Medicine Copenhagen, Department of Orthopaedic Surgery Copenhagen University Hospital ‐ Bispebjerg and Frederiksberg Copenhagen Denmark; ^2^ Department of Clinical Medicine, Faculty of Health and Medical Sciences University of Copenhagen Copenhagen Denmark

**Keywords:** aging, angiotensin II type I receptor, biomarker, hypertrophy, neuromuscular system, neuroprotection, strength training

## Abstract

**Background:**

Sarcopenia represents a major clinical and societal challenge facing rapidly aging populations. Accessible and specific biomarkers represent valuable tools, both in diagnosis and assessing the efficacy of therapeutic interventions. C‐terminal agrin fragment (CAF) is the most commonly used blood‐based biomarker of neuromuscular junction degradation in aging, inactivity and disease, but large unexplained interindividual variation exists, limiting its diagnostic and prognostic value. Exercise and medication may explain some of this variation. The aim of this study was to investigate the influence of a single bout (1EX) or 48 bouts (48EX) of heavy resistance exercise (EX), with or without angiotensin II type I receptor blocker (losartan (LOS)) supplementation, on serum CAF levels in healthy older men.

**Methods:**

Eighty‐three healthy, normotensive older men were enrolled in one of two randomized placebo (PLA) controlled trials. 1EX: 25 participants (EX ± LOS), with a mean age of 70 ± 7 years, had blood drawn before and after (4.5 h, Days 1, 4 and 7) a single bout of unilateral heavy resistance exercise of the quadriceps muscles. 48EX: at baseline, and after 8 and 16 weeks of a progressive heavy resistance exercise program, 58 participants (LOS‐EX, *n* = 20; LOS‐SED, n = 20; PLA‐EX, *n* = 18), with a mean age of 72 ± 5 years, had blood drawn, and specific force (strength per unit mass) was measured by dynamometer and magnetic resonance imaging of the quadriceps muscles. Serum CAF was measured by ELISA.

**Results:**

At baseline, CAF showed weak correlations with age and leg lean mass (both *R* ^2^ = 0.07, *p* < 0.05). With 48EX, specific force increased in both EX groups (LOS‐EX + PLA‐EX) by 13%–14% at 8 weeks and 14%–17% at 16 weeks (*p* < 0.0001), with no change in LOS‐SED (*p* > 0.05), confirming the efficacy of the 48EX program. Serum CAF increased in LOS‐EX and LOS‐SED by 4%–7% at 8 weeks and 7%–9% at 16 weeks (*p* < 0.005) respectively, with no change in PLA‐EX (*p* > 0.05). 1EX reduced CAF by 8% 1 day postexercise (*p* < 0.05), with no correlation to circulating creatine kinase levels (*p* > 0.05), a marker of muscle damage.

**Conclusions:**

Serum CAF was unaffected by 16 weeks of EX but increased by LOS supplementation. 1EX, performed with one leg, acutely lowered serum CAF, albeit with substantial interindividual variability. These findings collectively identify novel stimuli of serum CAF turnover—drug interaction and time from last exercise bout to blood sampling. These findings add value to CAF as a neuromuscular biomarker and highlight important experimental design aspects for future clinical studies.

## 1 Introduction

With aging, muscle strength and muscle power undergo greater declines compared to muscle mass [1], resulting in reduced specific muscle force (strength per unit mass) [2]. Although there are many underlying reasons for these discordant trajectories, muscle fibre denervation and destabilization of neuromuscular junctions play a key role [3, 4]. The consequences of these age‐related losses are severe. It has been estimated that about one in 10 above 65 years of age and one in four above 80 years of age develop the clinical diagnosis of sarcopenia, which is associated with impaired physical function, frailty and disability [5]. With imminent changes in the demographic composition of many western societies [S1], there is a socioeconomic need to define the most effective treatment strategies for maintaining muscle function and to identify readily accessible biomarkers that accurately and in a detailed manner can inform on this process.

Agrin is released from the axon terminals of the motor neuron and orchestrates the postsynaptic aggregation of the neuromuscular junction [S2]. At the neuromuscular junction, agrin can be inactivated by cleavage by the enzyme neurotrypsin, which leads to the release of a fragment from the C‐terminal end into the circulation [S3–S4]. This C‐terminal agrin fragment (CAF) is currently the most widely used blood‐based biomarker of neuromuscular junction instability in aging [6]. Increased cleavage of agrin, and consequently increased levels of circulating CAF, is indicative of neuromuscular junction disassembly [3, 7]. Accordingly, over the past 10 years, it has been shown that circulating CAF is higher in patients suffering from sarcopenia [8, 9, 10, 11, 12, 13], cachexia [14], heart failure [15], COPD [16], Alzheimer's disease [17], haemorrhage [18] and stroke [19], as compared to the blood levels in respective control populations.

While age‐related losses of muscle strength and power gradually progress throughout life, there is currently no clear consensus that CAF increases with aging *per se* [6]. In fact, two out of five studies including large groups of otherwise healthy individuals did not observe any differences in CAF levels between individuals of different ages [8, 20, 21, 22, 23]. Recently, Pratt et al. measured CAF in a cohort of 1000 men and women aged 18–87 and found a very weak (*r* = 0.08), albeit significant (*p* < 0.05), correlation between age and CAF levels [22]. Interestingly, the dataset also showed substantial interindividual variation, suggesting that parameters other than age per se are important for circulating CAF levels. As such, the relationship between CAF and indices of muscle mass [11, 12], strength [12, 24] and function [12, 25] has been explored in multiple studies, with findings generally showing weak to modest correlations [6]. Given that age and measures of physical function collectively explain less than half of the variation observed in CAF, other currently unrecognized variables must be involved. These may include diurnal variation, acute effects of exercise and potential interactions with medication. Medication use in particular is higher in older populations, and blood pressure lowering medication is one of the most commonly prescribed for individuals aged 45 and older. Interestingly, positive side effects beyond the primary action of the drug have been reported for angiotensin II receptor blockers (ARB), a class of blood pressure lowering medication that in 2023 was used by 25% of all citizens above 65 years of age in Denmark (www.medstat.dk). Although not entirely in agreement, rodent and human studies suggest protective effects of ARB use on muscle function, enabled through a growing number of proposed mechanisms [S5], including increased insulin‐like growth factor 1 signalling in mouse skeletal muscle [26]. It is therefore possible that ARB intake confers positive effects on neuromuscular junction integrity, manifested as reductions in CAF levels. We hypothesized, therefore, that ARB intake would explain some of the interindividual variation in serum CAF, reducing the levels either in combination with exercise training or independently through the proposed protective effects on neuromuscular function.

The observed link between CAF and measures of physical function suggests that physical activity and exercise play a role in the maintenance of neuromuscular junctions [27]. Currently, a total of 15 studies have investigated CAF responses to long‐term exercise interventions in humans [11, 19, 24, 25, 27, 28, 29, 30, 31, 32, 33, 34, 35, 36, 37]. From those studies, no clear conclusion can be drawn, as studies either find reduced [11, 19, 24, 27, 28, 29, 30, 31], unchanged [25, 33, 34, 35, 37] or even increased [32, 36] circulating levels of CAF by long‐term exercise. Importantly, the 15 published long‐term exercise intervention studies include vastly different study populations, such as frail and prefrail older adults [11, 25, 33, 34, 36], healthy older adults [24, 27, 28, 30, 31, 32] and patients suffering from COPD [29], stroke [19] or osteoarthritis [35], and they employ highly varied training regimes, such as specific rehabilitative programs [19], resistance/power [11, 24, 28, 32, 33, 37], dancing [27, 31], aerobic [29, 30, 33], combined aerobic and resistance [25, 27, 33, 34], skiing [35], TRX [36] or blood‐flow restriction [24, 30] training. Furthermore, five of the studies do not report CAF values for a nonexercising control group [11, 19, 27, 28, 29]. Finally, it is worth pointing out that there are no studies investigating the acute effects of exercise on circulating CAF, that only six out of the 15 exercise intervention studies specify the time from the last training bout until the blood sample was obtained [24, 28, 30, 31, 36, 37], that nine of the 15 studies state that the time of day for sampling was standardized [6, 19, 24, 28, 30, 31, 32, 35, 36] and that only three of the 15 studies provide any detail on medication use [19, 31, 37]. These inconsistencies and lack of detail may explain some of the interindividual variation and discrepancies among the findings of the long‐term intervention studies.

The aim of this study was twofold: firstly, to investigate changes in serum CAF concentration in response to long‐term (16 weeks) heavy resistance exercise with or without intake of a commonly used blood pressure lowering medication (ARB class and losartan (LOS)), and secondly, to explore the influence of a single session of heavy resistance exercise on serum CAF levels in the hours and days after exercise. We hypothesized that long‐term heavy resistance exercise, leading to increases in muscle strength and mass, would reduce levels of CAF, and that CAF would be lowered by LOS medication. Further, we wanted to map the time course of serum CAF fluctuations in the hours and days after a single session of heavy resistance exercise training.

## 2 Methods

### 2.1 Study Design and Participants

The study was approved by the Committees on Health Research Ethics for the Capital Region of Denmark (reference: H‐3‐2012‐081 and H‐15005761) and conformed to the standards set by the Declaration of Helsinki. Written informed consent was secured from all participants prior to enrolment. Participants were males living in the greater Copenhagen area, who were ≥ 64 years of age, normotensive and with a body mass index (BMI) between 19 and 34 kg/m^2^. Exclusion criteria included smoking, various diseases, the use of anticoagulative or blood pressure medication and regular exercise. Various diseases included cancers, organ dysfunctions, kidney/liver/connective tissue diseases and ulcers. All participants were sedentary or moderately active through walking and cycling for transportation, corresponding to tiers 0 and 1 in the classification framework by McKay et al. [38].

The original purpose of the study was to probe the effect of ARB (LOS) intake on skeletal muscle responses to acute and long‐term heavy resistance exercise [39, 40]. In both studies, participants were randomized, based on age, thigh lean mass and their angiotensin‐converting enzyme (ACE) genotype, into either LOS with exercise (LOS‐EX), placebo with exercise (PLA‐EX) or LOS without exercise (LOS‐SED, ‘sedentary’, only in long‐term study). In total, 25 and 58 participants completed all tests in the acute and long‐term parts, respectively, and were included for analyses. In the acute study, henceforth referred to as 1EX, participants performed a single bout of heavy resistance exercise, designed to induce a strong stimulus of the muscles, on Day 0, and had blood samples taken immediately before and 4.5 h, 1 day, 4 days and 7 days after. In the long‐term part of the study, henceforth referred to as 48EX, participants in LOS‐EX and PLA‐EX completed 48 sessions, over 16 weeks, of heavy resistance exercise, while LOS‐SED continued their normal lifestyle. Testing was conducted before (Week 0), midway (Week 8) and after (Week 16) and included blood sampling, magnetic resonance imaging (MRI) and assessment of maximal voluntary contraction (MVC) force.

### 2.2 Drug Administration

Participants ingested one pill containing LOS or placebo per day. LOS and placebo pills were identical in appearance, were produced by the local hospital pharmacy and delivered in blinded containers. In 1EX, 100 mg of LOS (or placebo) was ingested from Day −10 until Day 7. In 48EX, participants ingested 50 mg of LOS (or placebo) per day for the first week and then increased to 100 mg per day for the remainder of the study. Blood pressure was monitored during the study [39, 40].

### 2.3 Long Term Heavy Resistance Exercise—48EX

Participants performed 48 (range 44–53) supervised training sessions across 16 weeks, equivalent to three weekly training sessions. Following warm‐up on a stationary bicycle, the participants performed three mandatory exercises for the lower body (horizontal leg press, seated leg extension and seated leg curl) and were offered to perform two additional upper body exercises (pulldown and shoulder press), which all participants decided to do. The 16‐week training program was separated into six phases with gradual increases in load intensity (67% of one‐repetition maximum (1RM) to 86% of 1RM) and in the number of sets (three to five) and reductions in the number of repetitions (15 to six). The 1RM in leg extension, leg press and leg curl were assessed six times, immediately before the following sessions: first (pre), seventh, 16th, 25th, 34th and 43rd (post). The result of the 1RM test was used to tailor the load intensity for the subsequent training sessions; although importantly, loading was also continuously adjusted to ascertain a high level of exertion in every training set. All training performed was logged in individual training diaries, including the number of sets, repetitions and load used, as well as machine settings [39].

### 2.4 Acute Bout of Heavy Resistance Exercise—1EX

Following a warm‐up on a stationary bicycle, participants performed a unilateral 1RM test in a leg extension machine (TechnoGym, Cesena, Italy) with their dominant leg. Afterwards, participants performed, in the same setup, five sets of 12 concentric repetitions at 70% of 1RM followed by four sets of six eccentric repetitions at 110% of 1RM, with 2‐min rest between sets [40].

### 2.5 Muscle Mass and Muscle Strength

Participants in 48EX underwent MRI, a dual‐energy X‐ray absorptiometry (DEXA) scanning and evaluation of MVC. Both thighs were MRI scanned at the radiology department of Hilleroed Hospital (Copenhagen, Denmark) using a Philips Ingenia 3.0 T scanner. The slice closest to 50% femur length was used, and the cross‐sectional area (CSA) of vastus lateralis (VL), rectus femoris (RF), vastus medialis (VM) and vastus intermedius (VI) was determined by manually delineating the contours of the muscles in OsiriX 8.5 (Pixmeo SARL, Bernex, Switzerland). All scans were analysed by the same person (blinded to group and timepoint). MVC was assessed isokinetically (60°/s) and isometrically (at 70° knee angle, 0° equal straight leg) in a dynamometer (Kinetic Communicator, model 500−11; Chattecx, Chattanooga, TN). All tests were led by the same person (blinded to group). Both MRI and MVC data have been published elsewhere [39]. A DEXA scan was performed at baseline, using a Lunar DPX‐IQ scanner (GE‐Healthcare), to determine whole body and leg lean mass (LegLM). Specific force, a measure of neuromuscular function, was assessed by dividing indices of muscle strength (isokinetic and isometric MVC) by indices of muscle mass (quadriceps CSA and LegLM), both at baseline and as delta values throughout the intervention.

### 2.6 Blood Sampling

Blood samples were taken by venipuncture, allowed to coagulate at room temperature for 15–30 min and then centrifuged at room temperature for 10 min at 4000 rpm. Serum was aliquoted into Eppendorf tubes (500 μL) and stored at −80°C. In 1EX, samples were taken immediately before and 4.5 h, 1 day, 4 days and 7 days after the acute exercise bout. In 48EX, blood samples taken at Weeks 8 and 16 were obtained 2 days after the last exercise session. All samples, except the +4.5 h in 1EX, were taken after an individually standardized breakfast and before lunch (between 08.00 and 12.00 in the morning), within an hour of each other for each participant. Blood mononuclear cells were isolated in a BD Vacutainer Mononuclear cell preparation tube (Becton‐Dickinson, Franklin Lakes, NJ) for ACE genotyping, as previously described [39].

### 2.7 Creatine Kinase (CK)

CK concentrations were determined in 1EX on a Cobas 8000 (Roche Diagostics GmbH, Mannheim, Germany). These data have previously been reported for the LOS and placebo groups separately [40].

### 2.8 Enzyme‐Linked Immunosorbent Assay (ELISA) Analysis of Serum CAF

CAF serum concentration was analysed using the Human Agrin SimpleStep ELISA Kit (ab216945, Abcam, Cambridge, United Kingdom), following the manufacturer's instructions. Samples were diluted one in five using the provided diluent and assessed in duplicate. All samples from one individual were analysed on the same plate, and samples from individuals of different groups (SED and EX) were mixed across all plates, thus negating plate effects. Optical density was recorded at 450 nm using a microplate ELISA reader (Thermo Scientific, Multiskan FC), and CAF concentration was determined from CAF standard curves (on each plate) and corrected for sample dilution. The average coefficient of variation (CV) between duplicate standards was 2.7 ± 2.4%, and the average CV between duplicate samples was 2.3 ± 2.5%. Batches 2101037971 and 2101034365 were used for the 1EX and 48EX samples, respectively.

### 2.9 Statistics

In 1EX, changes in CAF concentration were evaluated using a one‐way mixed‐effects model and Dunnett's post hoc test with time as independent factor. In 48EX, changes in CAF concentration were evaluated using two‐way mixed‐effects model with time (pre, mid and post) and group (LOS‐EX vs. PLA‐EX vs. LOS‐SED) as independent factors and Tukey's post hoc test. The influence of training (LOS‐EX vs. LOS‐SED) and LOS medication (LOS‐EX vs. PLA‐EX) was examined in two separate tests. Grubb's test was used to identify outliers. Pearson's correlation analysis was performed for both baseline data and delta changes. Specifically, CAF concentration was correlated with age, BMI and several indices of muscle mass and strength at baseline, and delta (pre to mid, pre to post) CAF concentration was correlated with delta muscle CSA from MRI and MVC. Data are shown as means ± standard error of the mean unless otherwise stated. Graphs and statistical analyses were conducted in GraphPad Prism (v. 10, GraphPad Software Inc., La Jolla, CA); statistical significance was set at *p* < 0.05, and trends (*p* < 0.1) are also indicated.

## 3 Results

### 3.1 Participant Characteristics

Participant characteristics are provided in Table 1. There were no significant differences in any variable between participants in 1EX and 48EX, nor between the groups within 1EX or within 48EX. Systolic and diastolic blood pressures were lowered at the group level in groups that received LOS [39, 40].

**TABLE 1:** Participant characterization in acute and long‐term study. Data shown has means and standard deviations with ranges. Abbreviations: 1RM, one‐repetition maximum; BMI, body mass index; MVC, maximal voluntary contraction.

	Acute						Long term							
	LOS‐EX (*n* = 12)		PLA‐EX (*n* = 13)		All (*n* = 25)		LOS‐SED (*n* = 20)		LOS‐EX (*n* = 20)		PLA‐EX (n = 18)		All (*n* = 58)	
	Mean ± SD	Range	Mean ± SD	Range	Mean ± SD	Range	Mean ± SD	Range	Mean ± SD	Range	Mean ± SD	Range	Mean ± SD	Range
Age (year)	69 ± 4	66–78	71 ± 9	64–90	70 ± 7	64–90	72 ± 6	66–85	71 ± 4	66–83	72 ± 6	65–83	72 ± 5	65–85
Height (cm)	182 ± 5	172–189	177 ± 4	172–184	180 ± 5	172–189	179 ± 7	161–190	179 ± 6	163–191	178 ± 8	162–188	178 ± 7	161–191
Weight (kg)	86 ± 7	74–97	79 ± 11	67–98	82 ± 10	67–98	83 ± 11	62–102	86 ± 10	69–108	83 ± 12	57–98	84 ± 11	57–108
BMI (kg/m^2^)	26 ± 2	23–29	25 ± 3	21–31	26 ± 3	21–31	26 ± 3	21–32	27 ± 3	20–31	26 ± 3	19–33	26 ± 3	19–33
Unilateral leg ext. 1RM (kg)	60 ± 13	33–80	53 ± 15	23–82	56 ± 14	23–82	—	—	—	—	—	—	—	—
Bilateral leg ext. 1RM (kg)	—	—	—	—	—	—	—	—	83 ± 19	60–143	85 ± 20	48–130	—	—
Knee extension MVC (Nm)	—	—	—	—	—	—	194 ± 41	136–279	198 ± 42	126–298	194 ± 57	75–270	195 ± 46	75–298

### 3.2 Baseline Levels of CAF and Indices of Muscle Mass and Strength

**FIGURE 1:**
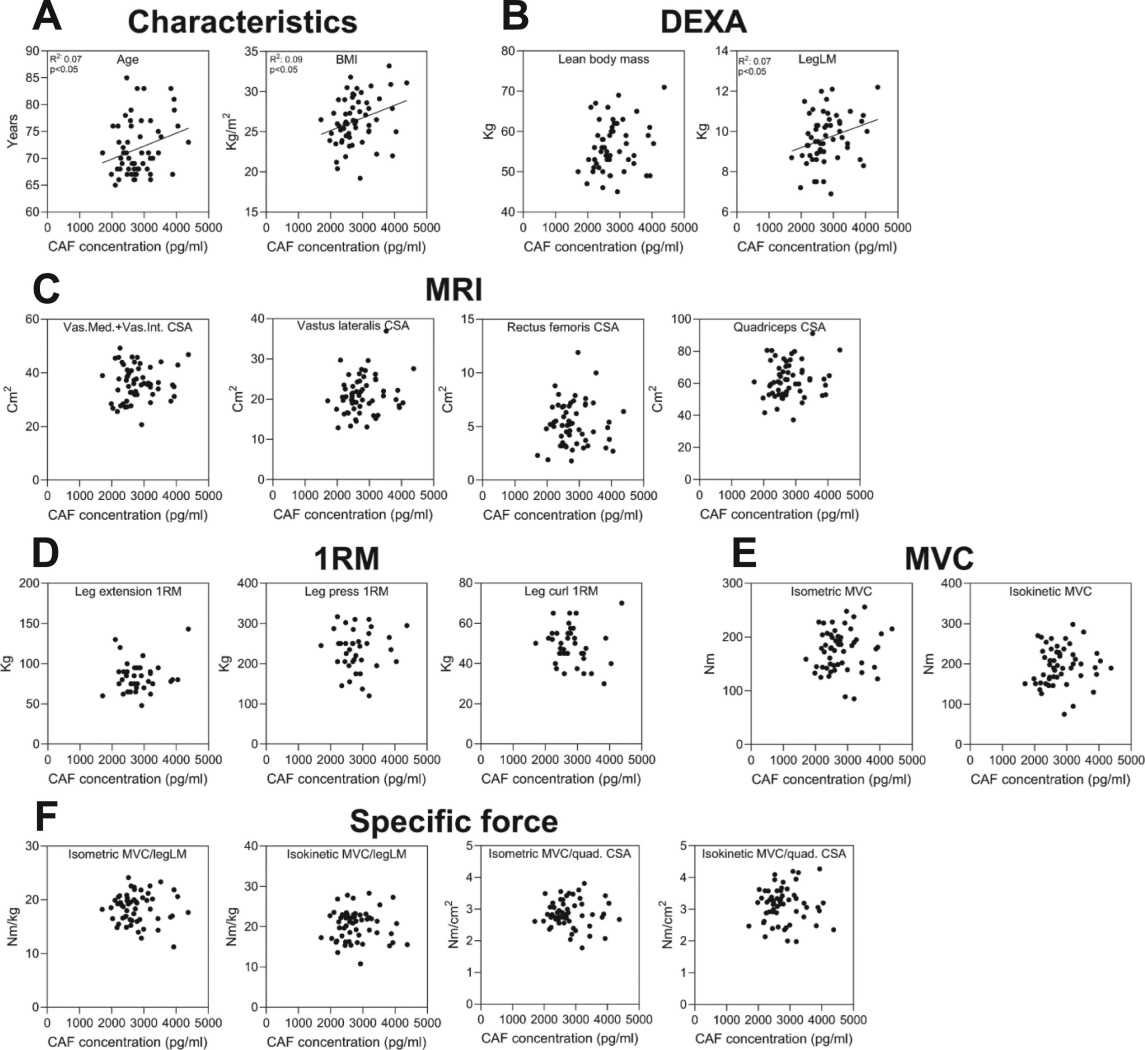
CAF is weakly correlated with age and leg muscle mass. Correlations in 48EX at baseline between CAF and participants characteristics (A), muscle mass measured by DEXA (B) and MRI (C), strength measured as 1RM (D) and MVC (E) and specific force (F). Data were analysed by Pearson's correlation analysis. *R* ^2^ value and *p* values are written for significant correlations (*N* = 58). Abbreviations: CAF, C‐terminal agrin fragment; BMI, body mass index; 1RM, one‐repetition maximum; LegLM, leg lean mass.

In 48EX at baseline, weak correlations were observed between CAF and age (*R* ^2^ 0.07, *p* < 0.05), BMI (*R* ^2^ 0.09, *p* < 0.05) and LegLM (*R* ^2^ 0.07, *p* < 0.05) (Figure 1A,B). There were no correlations between CAF and several MRI‐based measures of muscle mass, muscle strength or specific force (Figure 1C–F). CAF levels did not differ between ACE genotypes (*p* > 0.05, Figure S1).

### 3.3 The Impact of Long‐Term Heavy Resistance Exercise on Levels of CAF—48EX

**FIGURE 2:**
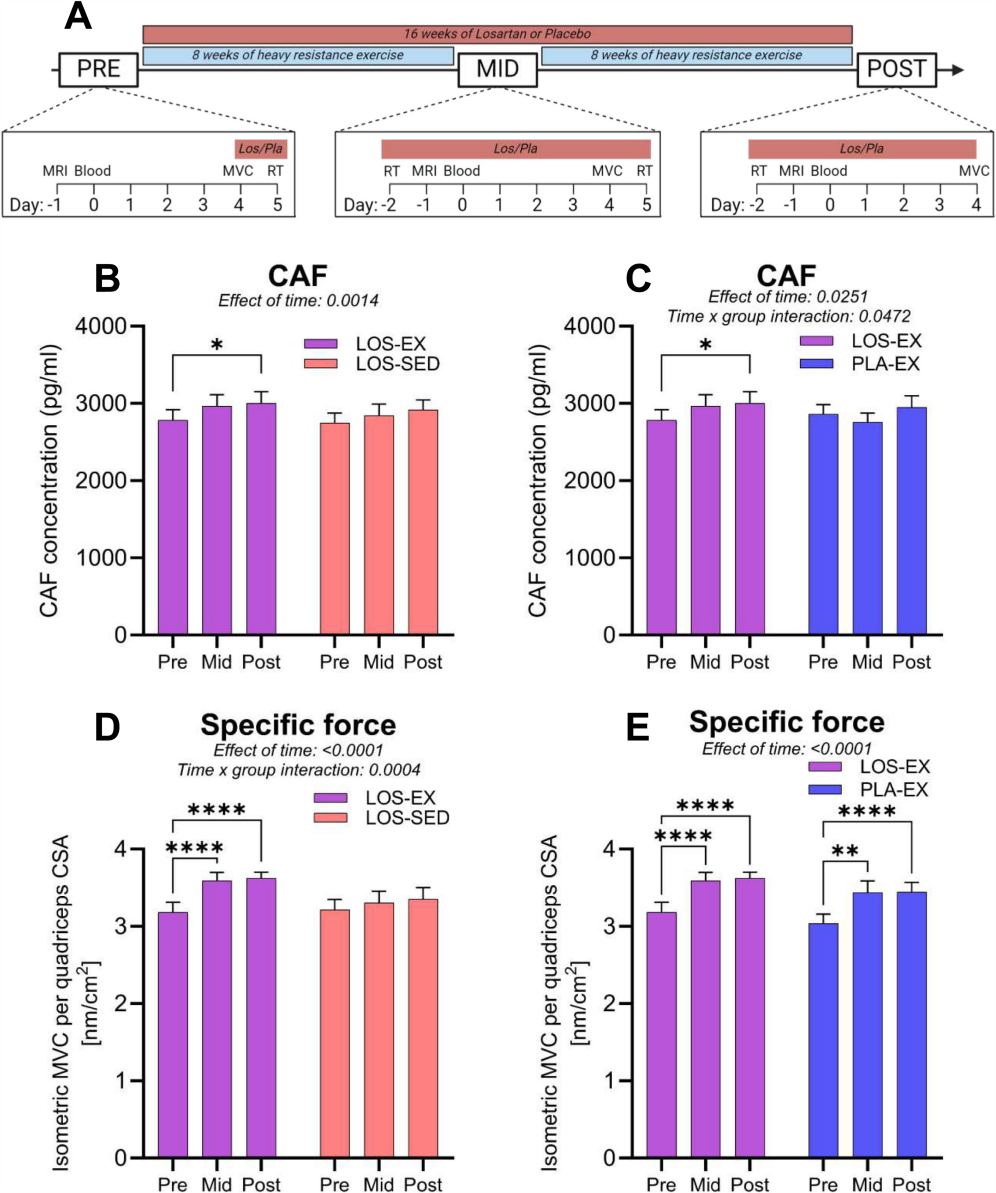
CAF is unaffected by long‐term heavy resistance exercise in healthy older men—48EX. (A) Experimental setup of the long‐term part of the study. Measurements were made before (pre), midway through (mid) and after (post) 16 weeks of heavy resistance exercise. (B, C) CAF measured before, midway through and after the 16‐week intervention in LOS‐EX (purple, *n* = 20), LOS‐SED (red, *n* = 20) and PLA‐EX (blue, *n* = 18). Data are shown as mean ± SEM and were analysed by mixed‐effects model (group × time), with main effects and interactions indicated, and Tukey's post hoc test was used. * indicates *p* < 0.05. (D, E) Specific force measured before, midway through and after the 16‐week intervention in LOS‐EX (purple, *n* = 20), LOS‐SED (red, *n* = 20) and PLA‐EX (blue, *n* = 18). Data are shown as mean ± SEM and were analysed by mixed‐effects model (group × time), with main effects and interactions indicated, and Tukey's post hoc test was used. ** indicates *p* < 0.001, and **** indicates *p* < 0.0001. Abbreviations: RT, resistance training; LOS, losartan; PLA, placebo; MRI, magnetic imaging resonance; CAF, C‐terminal agrin fragment; MVC, maximal voluntary contraction; CSA, cross‐sectional area.

A main effect of time was observed for CAF to increase during the intervention for both the combination of LOS and exercise (LOS‐EX) as well as when medication was taken without any exercise (LOS‐SED) (*p* < 0.005, Figure 2B). A time × group interaction was observed for LOS‐EX and PLA‐EX (*p* < 0.05), with post hoc tests showing an increase at 16 weeks only in LOS‐EX (*p* < 0.05, Figure 2C). Individual responses ranged from −18% to 60%, −10% to 39% and −16% to 27%, in LOS‐EX, LOS‐SED and PLA‐EX, respectively. Main effects of ACE genotype were observed for delta changes (pre to post) in CAF in LOS‐EX and LOS‐SED (*p* < 0.05) and LOS‐EX and PLA‐EX (*p* = 0.0941, Figure S1).

Specific force, defined as isometric knee extension MVC per quadriceps CSA, increased from pre to mid and pre to post in LOS‐EX and PLA‐EX (*p* < 0.0001), but not in LOS‐SED (Figure 2D,E). Out of 42 correlations, only specific force changes from pre to mid in LOS‐SED (*R* ^2^ 0.21, *p* < 0.05) and VL CSA changes from pre to post in LOS‐EX (*R* ^2^ 0.2, p < 0.05) were correlated with changes in CAF concentration (Figure S2).

The 1RM in leg extension and leg curl was increased by the seventh training session (*p* < 0.0001) and had increased by 64% and 32%, respectively, after 43 training sessions (*p* < 0.0001, Figure S3).

### 3.4 The Impact of Acute Heavy Resistance Exercise on Levels of CAF—1EX

**FIGURE 3:**
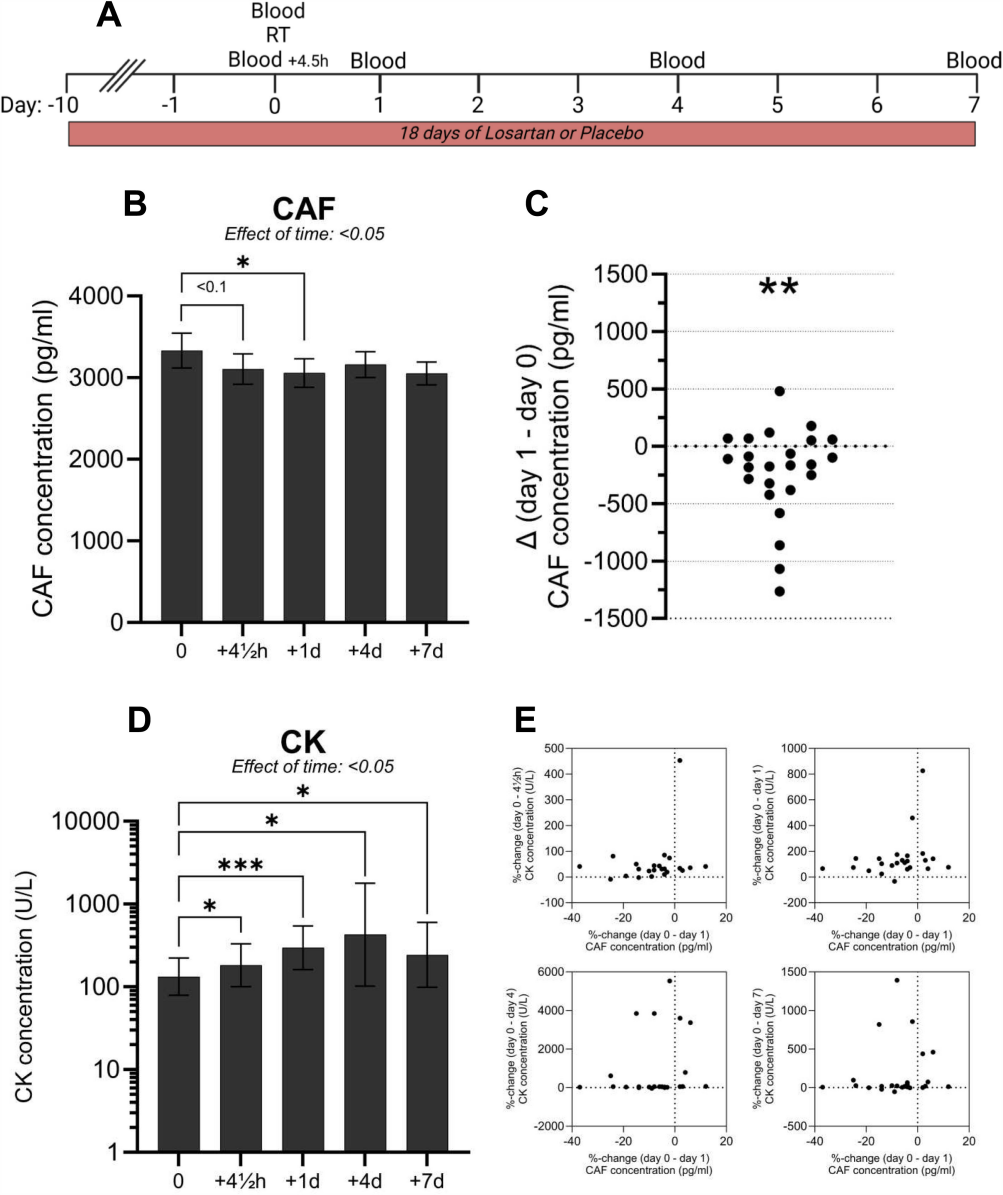
CAF is reduced by acute heavy resistance exercise in healthy older men—1EX. (A) Experimental setup of the acute part of the study. (B) CAF concentration before and 4.5 h, 1, 4 and 7 days following a bout of heavy resistance exercise (unilateral leg extensions). Data are shown as mean ± SEM and were analysed by mixed‐effects model (time), with main effect indicated, and Dunnett's post hoc test was used. * indicates < 0.05. Using Grubb's test, one subject's values identified as outliers for all time points except Day 7. (C) Individual CAF concentration delta values (Day 1–Day 0). One outlier with very high values at all time points except Day 7 was removed. Data were analysed by paired, two‐tailed *t*‐test. ** indicates < 0.01. (D) CK concentration before and 4.5 h, 1, 4 and 7 days following a bout of heavy resistance exercise. Data are shown as geometric means and geometric standard deviations on a logarithmic scale and were analysed by mixed‐effects model (time), with main effect indicated, and Dunnett's post hoc test was used. * indicates < 0.05, and *** indicates < 0.001. (E) Correlation analyses (Pearson's) between %‐change CAF (Day 0–1) and %‐change CK (Day 0 to 4½ h, 1 day, 4 days and 7 days). Abbreviations: LOS, losartan; PLA, placebo; CAF, C‐terminal agrin fragment; CK, creatine kinase.

CAF was reduced by 8.2% at the group level 1 day after an acute bout of exercise (*p* < 0.05, Figure 3B), with individual responses ranging from −37% to 12%. The values of one participant, who was also the oldest participant, were identified using Grubb's test as outliers at all time points except Day 7. When this participant was removed from the analysis, the statistical outcome of the one‐way mixed‐effects model showed a tendency for a main effect of time (*p* = 0.074). To control for potential variation at baseline, a direct comparison between Day 0 and 1 (delta values) by *t*‐test without the outlier revealed a clear reduction in circulating CAF levels (*p* < 0.01, Figure 3C). Noticeably, seven out of 25 individuals had delta values > 0, indicating the presence of response variability.

LOS intake had no effect on CAF during the 7‐day intervention (Figure S4). The acute exercise bout led to an increase in circulating CK, a surrogate marker of muscle damage, by 4.5 h (*p* < 0.05) and 1 day (*p* < 0.0005), peaked by Day 4 (*p* < 0.05), and remained elevated at Day 7 (*p* < 0.05, Figure 3D). There was no correlation between ΔCAF (Day 0–Day 1) and ΔCK at any time point (Figure 3E).

## 4 Discussion

The main finding of this study is that serum levels of CAF, a widely used marker of neuromuscular junction instability, are not reduced by 16 weeks of heavy resistance exercise, despite a significant increase in specific force (strength per unit mass). This suggests a decoupling between training‐induced improvements in physical function and changes in the most widely used biomarker of neuromuscular junction instability. In contrast to our hypothesis, we found that a commonly used ARB class of blood pressure‐lowering medicine (LOS) led to a 7%–9% increase in CAF levels in 16 weeks. Further, this study also showed that a single bout of strength exercise acutely reduced CAF levels in most participants, but that this effect, when repeated over time, could not counteract the increase in circulating CAF caused by 16 weeks of ARB intake. These findings add to the value of CAF in aging research by explaining some of the large interindividual variation in CAF levels reported in the literature.

At baseline, few and weak correlations were observed between CAF and key characteristics or measures of muscle mass and muscle strength, which is in alignment with a recent cohort study with 1000 men and women aged 18–87 [22]. Some studies have reported weak to moderate correlations between CAF and measures of muscle mass, and to a lesser degree measures of muscle strength [6]. This indicates that levels of CAF are dictated by yet unknown factors beyond markers of muscle mass and quality. If CAF, as generally believed, specifically informs on the structural integrity of neuromuscular junctions, it can be expected to decline following a long‐term exercise intervention successfully inducing hypertrophy and strength gains. This was not observed with 16 weeks training in the PLA‐EX or the LOS‐EX group. This is surprising given that we have previously analysed muscle samples of these individuals and observed positive exercise‐induced changes in tissue markers of innervation status [41] and hypertrophy [39]. However, we observed a clear increase over time in circulating CAF in the groups that received the ARB class of blood‐pressure lowering medication, LOS.

The lack of decrease in CAF with a whole‐body exercise intervention that caused significant increases in muscle strength and muscle mass questions how changes in CAF with exercise should be interpreted, at least in healthy older men. Our finding is in contrast to some prior exercise studies finding reductions in CAF concentration among healthy older individuals [24, 27, 28, 30, 31]. Among these, two used dancing exercise [27, 31] and two used blood‐flow restriction training [24, 30], suggesting that reductions in CAF concentration can be achieved through different modes of exercise. Additionally, given that the participants in the present study were healthy (normotensive, no blood pressure medication at baseline), and that the baseline CAF values were relatively low, the potential for further therapeutic improvement in CAF by exercise might be limited. The potential for improvement might therefore be greater in frail older adults with higher baseline CAF values [11, 25, 33, 34].

It was unexpected to observe such a marked effect of blood pressure medication upon circulating CAF concentration. An increase in CAF with ARB intake indicates a direct negative effect on motor neurons and/or neuromuscular junctions, potentially by binding to receptors that are found in many different tissues, including brain neurons [S6]. In support of this interpretation, Qaisar et al. showed, in an uncontrolled study, that treatment of hypertensive Alzheimer's patients for 1 year with various antihypertensive drugs (including ARBs), increased circulating levels of CAF [17]. Importantly, this interpretation is refuted by three main points. First, any negative effect of LOS medication on muscle function is not supported by the data in the present study on muscle mass and muscle strength, where specific force was statistically unchanged (and numerically increased) in the group that received LOS without training (LOS‐SED). Second, another study by Qaisar et al. showed no effect of a 1‐year treatment with ARB in hypertensive COPD patients on CAF [16]. Third, there is a vast amount of literature in humans and rodents that link ARBs to protective effects on muscle function, as discussed elsewhere [S5]. Instead, we suggest an alternative interpretation. ARB's mechanism of action is complex and involves multiple organs. Key to its effect is inhibition of angiotensin II binding to its receptor (Subunit I), which prevents vasoconstriction and increases renal perfusion. Agrin, which gives rise to CAF, is, in addition to its expression at the neuromuscular junction in skeletal muscle, highly expressed in kidney tissue [S7], and CAF levels have been tightly linked to kidney function [21]. The effect of LOS on blood pressure in the present study makes it likely that the kidney is the source of increased CAF. We also observed an influence of ACE genotype on changes in CAF. The heterozygous ACE genotype (ID) has previously been linked to LOS responsiveness in diabetic nephropathy [42], and our results highlight a novel interaction between LOS usage and ACE genotype in CAF levels.

An important secondary finding of the present study was, in contrast to our hypothesis, that a single bout of heavy resistance exercise reduced circulating levels of CAF after 1 day at the group level, with some interindividual variability in the response. This is a rather surprising finding that has important implications for future exercise studies on neuromuscular junction integrity. Although the exercise was carried out only in one leg, the exercise resulted in significant increases in the level of circulating CK until 7 days postexercise, increased the number of satellite cells at Days 4 and 7, led to macrophage infiltration and resulted in a marked response at the mRNA level [S5, S8]. This, collectively, indicates a substantial activation of the thigh muscles, beyond anything these sedentary participants were accustomed to, and thus it is likely that some degree of muscle damage occurred. As such, it was expected that if CAF were to change with acute exercise, it would increase in circulation, stemming from the damaged fibres whose innervation may have been perturbed. Instead, CAF was reduced after 1 day and was back to baseline levels at 4 days, essentially mirroring the pattern of gene expression for some of the acetylcholine receptors (AChR) measured in the muscles of these participants [43]. We evaluated whether this acute effect was linked to muscle damage but observed no correlation between %‐change in CAF and CK. Given that neuromuscular junctions are highly plastic structures [S9] that adapt to various stimuli [S10], it can be theorized that the reduction in CAF concentration reflects a temporary strengthening of neuromuscular junctions by virtue of less cleavage by neurotrypsin. Since the exercise‐induced effect on serum CAF levels was gone by 4 days, it can be speculated that optimal neuroprotective exercise requires frequently repeated bouts of exercise that each have a protective effect for a certain period (1–3 days). Importantly, this acute systemic effect was observed following a very intense unaccustomed exercise bout performed with only one leg. Thus, it can be further speculated that less intense, whole‐body exercise could have similar effects. Perhaps the most important point, though, is that our findings have implications for both cross‐sectional and longitudinal exercise studies, as lack of standardization of blood sampling in relation to the last exercise bout could lead to inaccurate measures of CAF levels and therefore erroneous conclusions.

The study is limited by its inclusion of only healthy men, which may affect the generalizability of the findings to women and mobility‐impaired individuals. However, circulating levels of CAF are similar between men and women across all ages [22]. Additionally, the participants in this study represent a selected population, as they, despite not engaging in structured physical activity, did not have hypertension or use blood pressure‐lowering medication. It is also worth noting that, since we did not have a nonexercise, no‐drug group (PLA‐SED), we were unable to include all independent variables (time, drug and exercise) in a single statistical model (three‐way ANOVA). Finally, in 1EX, a unilateral exercise regimen was chosen to study the muscle's response to exercise, which may not have been optimal for assessing a circulating biomarker.

## 5 Conclusions

Serum CAF in normotensive, healthy older men is unaffected by 16 weeks of heavy resistance exercise but increased by 7%–9% with ARB medication intake, despite a marked training‐induced increase in specific muscle force, suggesting that there is not a tight coupling between muscle adaptations to training and circulating CAF. Finally, the study demonstrated that a single bout of unaccustomed strength exercise in itself acutely reduced CAF levels, but this effect, even when repeated over time, could not counteract the increase in circulating CAF induced by blood‐pressure lowering medication. Future studies involving CAF should account for blood pressure‐lowering medication usage.

## Ethics Statement

The authors certify that they comply with the Ethical Guidelines for authorship and publishing of the Journal of Cachexia, Sarcopenia and Muscle [S11].

## Conflicts of Interest

The authors declare no conflicts of interest.

## Supporting information


**Figure S1.** ACE genotype influences levels of CAF. (A) CAF measured before the 16‐week intervention in carriers of the DD (*n* = 16), DI (*n* = 28) and II (*n* = 14) genotypes. Data are shown as mean ± SEM and were analysed by mixed‐effects model (group), and Tukey’s post hoc test was used. (B) CAF delta values (pre to post) in carriers of the DD (*n* = 11), DI (*n* = 21) and II (*n* = 8) genotypes in LOS‐EX and LOS‐SED. Data are shown as mean ± SEM and were analysed by mixed‐effects model (group × genotype), with main effects and interactions indicated, and Tukey’s post hoc test was used. (C) CAF delta values (pre to post) in carriers of the DD (*n* = 11), DI (*n* = 21) and II (*n* = 8) genotypes in LOS‐EX and PLA‐EX. Data are shown as mean ± SEM and were analysed by mixed‐effects model (group × genotype), with main effects and interactions indicated, and Tukey’s post hoc test was used. Abbreviations: LOS, losartan; PLA, placebo; CAF, C‐terminal agrin fragment; DD, deletion/deletion; DI, deletion/insertion; II, insertion/insertion.
**Figure S2.** Correlations between %‐change in CAF in %‐change in 7 indices of muscle mass and strength, from pre to mid and from pre to post, for LOS‐EX (purple), LOS‐SED (red) and PLA‐EX (blue). *R* ^2^ value and *p* values are written for significant correlations. Abbreviations: LOS, losartan; PLA, placebo; CAF, C‐terminal agrin fragment; MVC, maximal voluntary contraction; CSA, cross‐sectional area; VM + VI, vastus medialis and vastus intermedius; VL, vastus lateralis; RF, rectus femoris.
**Figure S3.** Progression in 1RM for the two exercise groups combined (LOS‐EX and PLA‐EX, *n* = 38), expressed in kilograms (top row) and as percentages relative to the first test (bottom row) in leg extension (A) and leg curl (B), measured before the first, seventh, 16th, 25th, 34th and 43rd training session. Data are shown as mean ± SEM and were analysed by mixed‐effects model (time) and Dunnett’s post hoc test. In all cases, a significant effect of time of *p* < 0.0001 was observed. **** indicates *p* < 0.0001. Abbreviations: 1RM, one‐repetition maximum.
**Figure S4.** CAF concentration before and 4.5 h, 1, 4 and 7 days following a bout of heavy resistance exercise (unilateral leg extensions) in LOS‐EX (purple, *n* = 12) and PLA‐EX (blue, *n* = 13). Data are shown as mean ± SEM and were analysed by mixed‐effects model (time × group), with main effect indicated, and Dunnett’s post hoc test was used. Abbreviations: LOS, losartan; PLA, placebo; CAF, C‐terminal agrin fragment.

**Data S1**. Supporting Information.
