# Supplementary material for: Serum C‐Terminal Agrin Fragment With Acute and Long‐Term Exercise and Angiotensin II Type I Receptor Blockade
Source: J Cachexia Sarcopenia Muscle. 2025 Jun 4;16(3):e13832. doi: 10.1002/jcsm.13832 (PMC12134776; doi:10.1002/jcsm.13832)

Supplemental figure 1

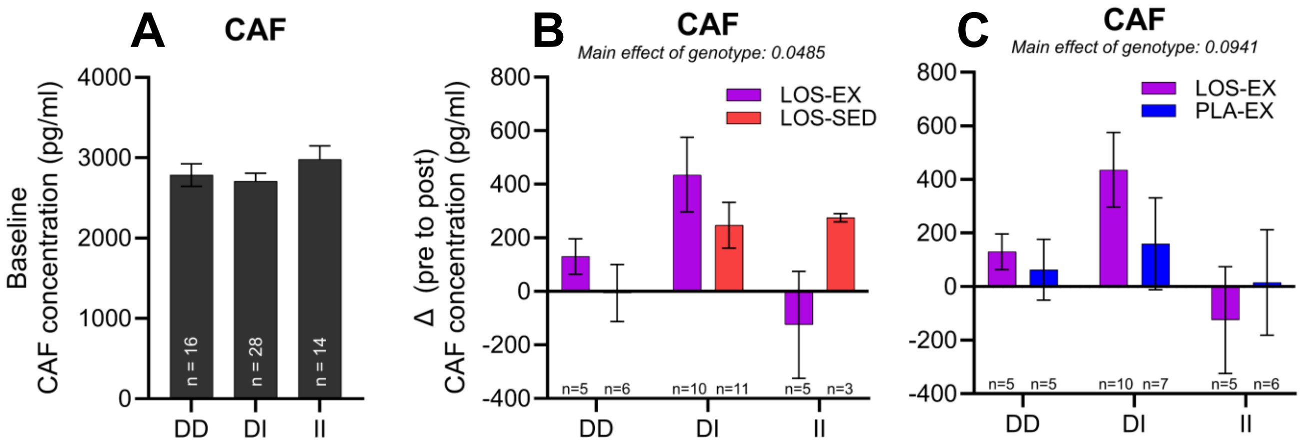

Supplemental figure 2

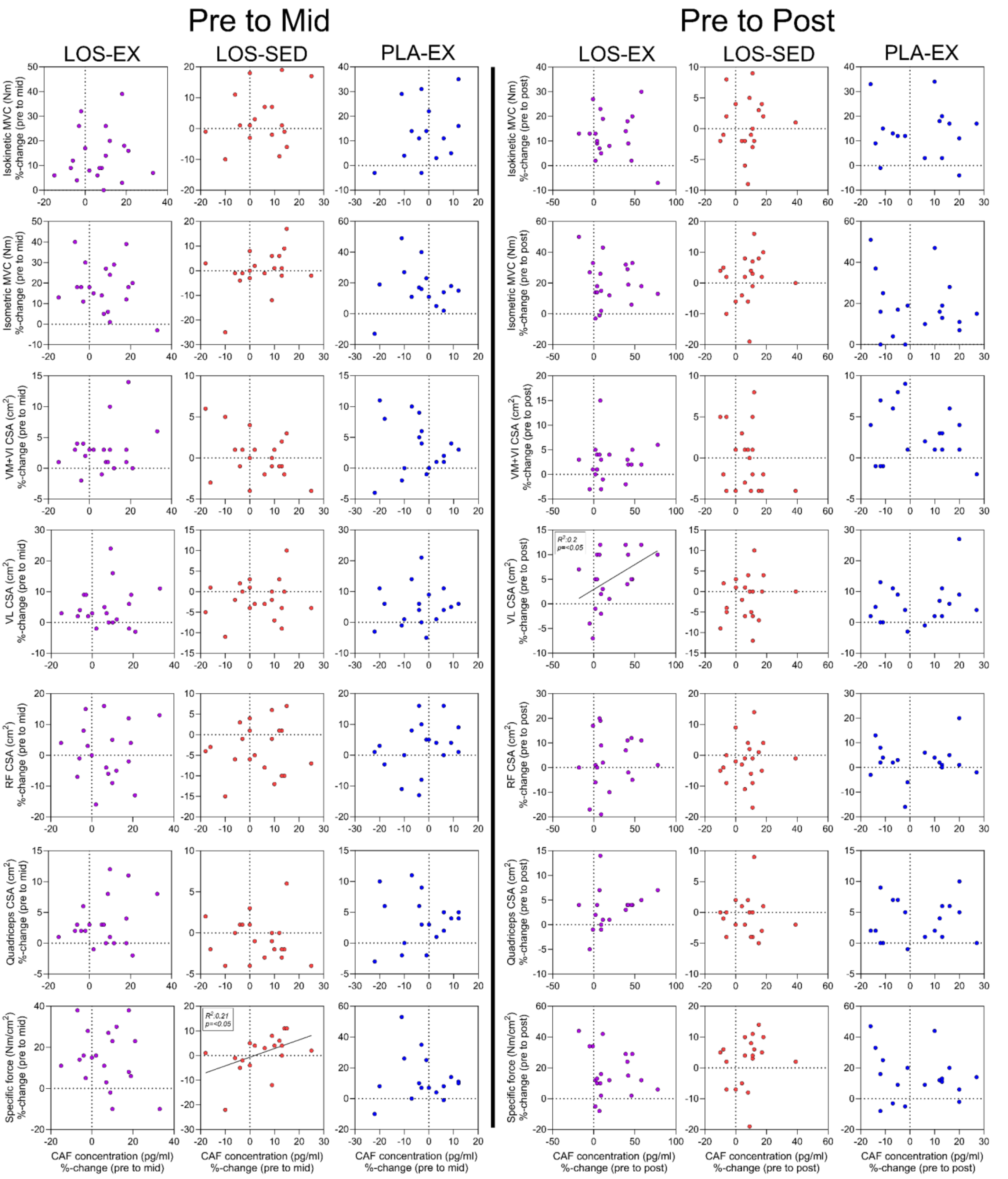

Supplemental figure 3

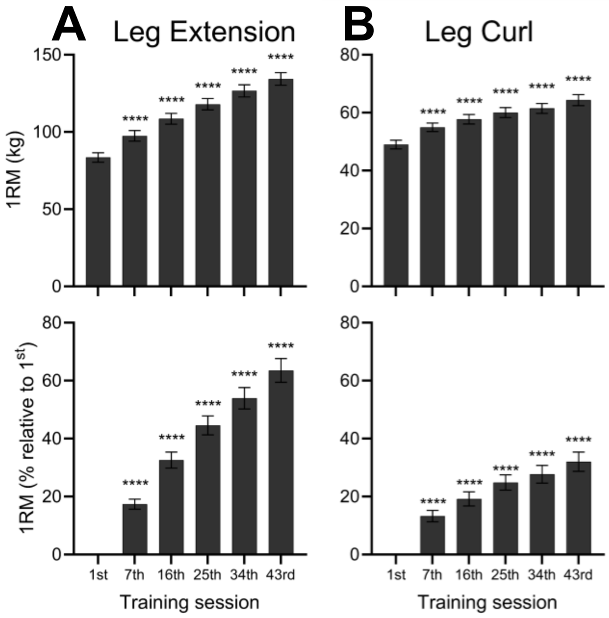

Supplemental figure 4

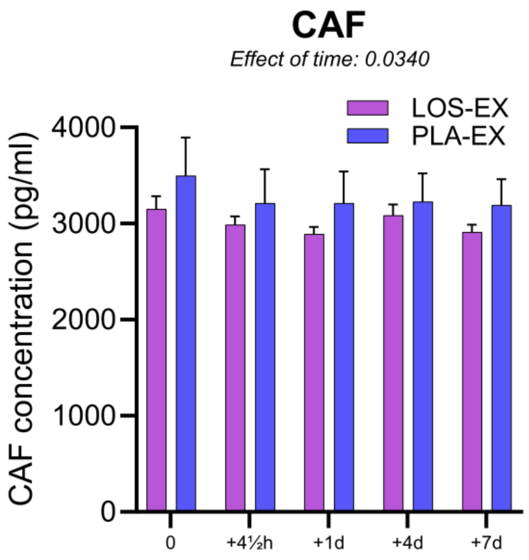

Supplement: Supplementary file 1 — Figure S1. ACE genotype influences levels of CAF. (A) CAF measured before the 16‐week intervention in carriers of the DD (n = 16), DI (n = 28) and II (n = 14) genotypes. Data are shown as mean ± SEM and were analysed by mixed‐effects model (group), and Tukey’s post hoc test was used. (B) CAF delta values (pre to post) in carriers of the DD (n = 11), DI (n = 21) and II (n = 8) genotypes in LOS‐EX and LOS‐SED. Data are shown as mean ± SEM and were analysed by mixed‐effects model (group × genotype), with main effects and interactions indicated, and Tukey’s post hoc test was used. (C) CAF delta values (pre to post) in carriers of the DD (n = 11), DI (n = 21) and II (n = 8) genotypes in LOS‐EX and PLA‐EX. Data are shown as mean ± SEM and were analysed by mixed‐effects model (group × genotype), with main effects and interactions indicated, and Tukey’s post hoc test was used. Abbreviations: LOS, losartan; PLA, placebo; CAF, C‐terminal agrin fragment; DD, deletion/deletion; DI, deletion/insertion; II, insertion/insertion. Figure S2. Correlations between %‐change in CAF in %‐change in 7 indices of muscle mass and strength, from pre to mid and from pre to post, for LOS‐EX (purple), LOS‐SED (red) and PLA‐EX (blue). R 2 value and p values are written for significant correlations. Abbreviations: LOS, losartan; PLA, placebo; CAF, C‐terminal agrin fragment; MVC, maximal voluntary contraction; CSA, cross‐sectional area; VM + VI, vastus medialis and vastus intermedius; VL, vastus lateralis; RF, rectus femoris. Figure S3. Progression in 1RM for the two exercise groups combined (LOS‐EX and PLA‐EX, n = 38), expressed in kilograms (top row) and as percentages relative to the first test (bottom row) in leg extension (A) and leg curl (B), measured before the first, seventh, 16th, 25th, 34th and 43rd training session. Data are shown as mean ± SEM and were analysed by mixed‐effects model (time) and Dunnett’s post hoc test. In all cases, a significant effect of time of p < 0.0001 was observe [file JCSM-16-e13832-s001.pdf]
