# Supplementary material for: Serum C‐Terminal Agrin Fragment With Acute and Long‐Term Exercise and Angiotensin II Type I Receptor Blockade
Source: J Cachexia Sarcopenia Muscle. 2025 Jun 4;16(3):e13832. doi: 10.1002/jcsm.13832 (PMC12134776; doi:10.1002/jcsm.13832)
Supplement: Supplementary file 2 — Data S1. Supporting Information. [file JCSM-16-e13832-s002.docx]

S1. United Nations Department of Economic and Social Affairs, Population Division. World Population Prospects 2022: Summary of Results. 2022.

S2. Bezakova G, Ruegg MA. New insights into the roles of agrin. Nat Rev Mol Cell Biol 2003;4:295–308.

S3. Stephan A, Mateos JM, Kozlov SV, Cinelli P, Kistler AD, Hettwer S et al. Neurotrypsin cleaves agrin locally at the synapse. FASEB J 2008;22:1861–1873.

S4. Reif R, Sales S, Hettwer S, Dreier B, Gisler C, Wölfel J et al. Specific cleavage of agrin by neurotrypsin, a synaptic protease linked to mental retardation. FASEB J 2007;21:3468–3478.

S5. Kingsley J, Torimoto K, Hashimoto T, Eguchi S. Angiotensin II inhibition: a potential treatment to slow the progression of sarcopenia. Clin Sci (Lond) 2021;135:2503–2520.

S6. Sumners C, Alleyne A, Rodríguez V, Pioquinto DJ, Ludin JA, Kar S et al. Brain angiotensin type-1 and type-2 receptors: cellular location under normal- and hypertensive conditions. Hypertens Res 2020;43:281–295.

S7. Steubl D, Hettwer S, Vrijbloed W, Dahinden P, Wolf P, Luppa P et al. C-terminal agrin fragment--a new fast biomarker for kidney function in renal transplant recipients. Am J Nephrol 2013;38:501–508.

S8. Jensen SM, Bechshøft CJL, Heisterberg MF, Schjerling P, Andersen JL, Kjaer M et al. Macrophage Subpopulations and the Acute Inflammatory Response of Elderly Human Skeletal Muscle to Physiological Resistance Exercise. Front Physiol 2020;11:811.

S9. Mansilla A, Jordán-Álvarez S, Santana E, Jarabo P, Casas-Tintó S, Ferrús A. Molecular mechanisms that change synapse number. J Neurogenet 2018;32:155–170.

S10. Valdez G, Tapia JC, Kang H, Clemenson GD, Gage FH, Lichtman JW et al. Attenuation of age-related changes in mouse neuromuscular synapses by caloric restriction and exercise. Proc Natl Acad Sci U S A 2010;107:14863–14868.

S11. von Haehling S, Coats AJS, Anker SD. Ethical guidelines for publishing in the Journal of Cachexia, Sarcopenia and Muscle: update 2021. J Cachexia Sarcopenia Muscle 2021;12:2259–2261.
